# Cognitive Rehabilitation of Adaptive Behavior in Children with Neurodevelopmental Disorders: A Meta-Analysis

**DOI:** 10.1155/2018/5029571

**Published:** 2018-09-12

**Authors:** Si-nae Ahn, Sujin Hwang

**Affiliations:** ^1^Department of Occupational Therapy, Cheongju University, 298 Daesung-ro, Cheongju-si, Republic of Korea; ^2^Deparment of Physical Therapy, Division of Health Science, Baekseok University, 76 Munam-ro, Dongnam-gu, Cheonan-si, Chungnam, Republic of Korea

## Abstract

**Objectives:**

Negative behavioral problems often occur following the onset of neurodevelopmental disorders and have an overall impact on the affected children, specifically in terms of their social developmental level. In children, social development behavior has been shown to spontaneously mature over time with the cognitive therapy intervention effects. This study performed a meta-analysis to provide a statistical synopsis of the available evidence of social development behavioral changes following cognitive therapy in children with neurodevelopmental disorders.

**Methods:**

Data was collected from two online search engines, including EBSCOhost and PubMed, from January 1, 2006, to August 31, 2016, using the terms “cognition,” “cognitive function,” and “disease including neurodevelopmental disorder with DSM-5.” Two assessors searched the literature using independent inclusion criteria and evaluated the quality of results using the Jadad score. Six articles were chosen using the Comprehensive Meta-Analysis program (version 2.0).

**Results:**

Six articles reporting randomized controlled trial studies were included. The effective scores for improving adaptive behavior following cognitive therapy in children with neurodevelopmental disorder were 0.64. The effective score of adaptive behavior was significant in this study (*p* < 0.05). The results showed no significant statistical heterogeneity and publication bias.

**Conclusions:**

The findings of the meta-analysis suggest that cognitive interventions are effective at improving adaptive behavior associated with neurodevelopmental disorders.

## 1. Introduction

Neurodevelopmental disorders refer to developmental, cognitive function, motor function, verbal communication, social skill, and behavioral disorders. The growth and development of the central nervous system is affected by these disorders. The American Psychiatric Association categorizes ten neurodevelopmental disorders: intellectual disabilities, global developmental delay, communication disorders, autism spectrum disorder, attention-deficit/hyperactivity disorder, specific learning disorders, developmental coordination disorders, stereotypical movement disorders, tic disorders, and Tourette's syndrome [1].

Children affected by neurodevelopmental disorders often face difficulties in undergoing normal cognitive development, because of the occurrence of cognitive problems from an early stage of development [2]. As a result, affected children fail to achieve the expected developmental progress for their respective developmental ages. Neurodevelopmental disorders are also associated with poor social function and various other negative behavior aspects in school-age and adolescent children [3, 4]. Specifically, neurodevelopmental disorders are characterized by difficulties with social communication and interaction across contexts, as well as restricted and repetitive patterns of behavior aspects [5, 6].

There has been a marked increase in cognitive interventions that are aimed at improving the symptoms or features of neurodevelopmental disorders. However, there is no evidence to support the use of such cognitive therapies to treat symptoms of behavioral abnormalities associated with neurodevelopmental disorders. Several articles conclude that early and intensive interventions can lead to improvements in adaptive and communicative behaviors, as well as in social skills [7, 8].

Children with a neurodevelopmental disorder who receive occupational therapy during early childhood can perform a variety of tasks according to their respective cognitive abilities [9–11]. These studies have shown that children with neurodevelopmental disorders have a good prognosis with regard to their ability to participate in tasks when early cognitive treatments are received early. Specifically, cognitive therapy at the preschool level was found to improve the prognosis of behavioral outcomes in children with neurodevelopmental disorders [9].

According to Lucas et al., some occupational therapy interventions with a task-oriented framework are shown to likely improve overall motor outcomes in children with neurodevelopmental disorders but overall results are limited because of the low quality of available evidence [12]. Weston et al. studied meta-analytic and systematic appraisal of the literature investigating the effectiveness of cognitive behavioral therapy (CBT) when used with individuals who have autism spectrum disorder (ASD). Their results demonstrate that the CBT is an empirically validated treatment for use with people who have ASDs [13].

As mentioned previously, few studies have examined the effectiveness of certain types of cognitive interventions with neurodevelopmental disorders (Wright et al., 2013). Additionally, there is insufficient evidence to support the beneficial effects of cognitive therapy on individuals with neurodevelopment disorders, including disorders such as intellectual disabilities, global developmental delay (GDD), communication disorders, ASD, attention-deficit/hyperactivity disorder (ADHD), specific learning disorders, and developmental coordination disorder (DCD).

Adaptive behavior is viewed as what an individual does to effectively meet social/cultural standards of personal independence and social responsibility. Social skills represent behaviors which, in specific situations, predict important social outcomes for children and youth [14]. Adaptive behaviors are an essential part of occupational therapy in facilitating the development of growing children with neurodevelopmental disorders [14]. Therefore, there is a need to understand the best therapeutic method to support the utilization of adaptive behavioral strategies.

Meta-analysis is needed to determine whether or not cognitive therapy to positively change adaptive behavior was useful in children with neurodevelopmental disorders. Therefore, we conducted a comprehensive meta-analysis of the literature, which is aimed at investigating the effectiveness of cognitive therapy as a part of a comprehensive rehabilitation program focused on improving functional performance in individuals with neurodevelopmental disorders.

## 2. Method

### 2.1. Search Strategy

The search was performed in a stepwise manner between January 1, 2006, and August 31, 2016. A comprehensive search of electronic databases was completed on the following electronic sources using EBSCOhost and PubMed. The search keywords used were “disease including neurodevelopmental disorder with DSM-5” AND “cognition,” and “disease including neurodevelopmental disorder with DSM-5” AND “cognitive function.” The final search expression was as follows: “cognition” AND “Intellectual Disability,” “cognitive function” AND “Intellectual Disability,” “cognition” AND “Intellectual Developmental Disorder,” “cognitive function” AND “Intellectual Developmental Disorder,” “cognition” AND “Global Developmental Delay,” “cognitive function” AND “Global Developmental Delay,” “cognition” AND “Autism Spectrum Disorder,” “cognitive function” AND “Autism Spectrum Disorder,” “cognition” AND “Attention Deficit Hyperactivity Disorder,” “cognitive function” AND “Attention Deficit Hyperactivity Disorder,” “cognition” AND “Developmental Coordination Disorder,” “cognitive function” AND “Developmental Coordination Disorder,” “cognition” AND “Stereotypic Movement Disorder,” “cognitive function” AND “Stereotypic Movement Disorder,” “cognition” AND “Tic Disorders,” “cognitive function” AND “Tic Disorders,” “cognition” AND “Tourette Disorder,” “cognitive function” AND “Tourette Disorder.”

### 2.2. Filtering Procedure and Study Selection

**Figure 1:**
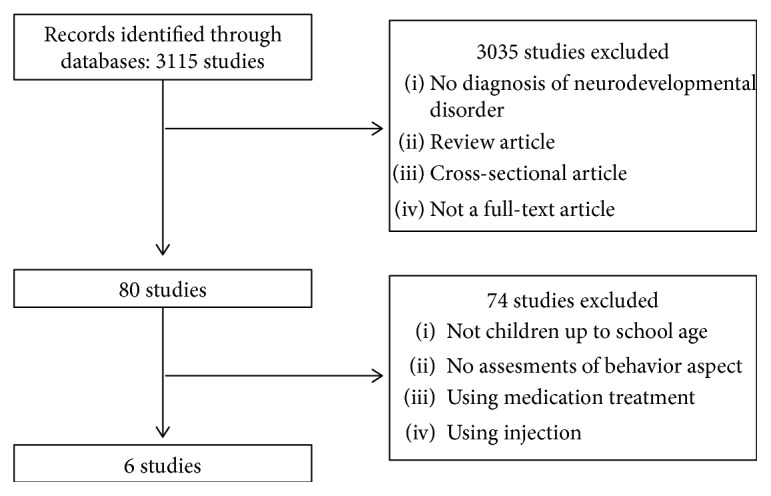
Study process.

This study searched for original articles that were published in academic journals, written in English, and met our inclusion and exclusion criteria. Using this method, a total of 3115 articles were identified in this study. Since it was not possible to automatically exclude animal trials in this search engine, this was done by hand. Abstracts of the remaining publications were scrutinized for eligibility by two independent assessors (i.e., the author Ahn and Hwang), using the inclusion criteria and exclusion criteria described below. Thus, a total of 80 articles were included in the full-text review by both assessors. The final list consisted of 6 studies (Figure 1).

### 2.3. Inclusion Criteria and Exclusion Criteria

First, titles and abstracts of studies were scanned for eligibility according to the following inclusion criteria: (a) population age range (i.e., children up to school-going ages under 13 years of age), (b) type of study design (i.e., randomized controlled trial), (c) targeting for neurodevelopmental disorder, (d) assessments of adaptive behavior, and (e) language in which article was written (English).

Secondly, titles and abstracts of studies were excluded based on the following criteria: (a) using medication treatment, (b) using injection, and (c) using acupuncture. Subsequently, the abstracts of the remaining subset were reviewed further.

### 2.4. Data Extraction

The following information was extracted: year of publication, first author, period, number of children with neurodevelopmental disorder in each group, diagnosis of a neurodevelopmental disorder, age of subjects enrolled, types of cognitive function intervention, and assessment tools of adaptive behavior. Data were extracted from eligible studies by two investigators acting independently, and any disagreement was resolved by consulting with two reviewers (Ahn and Hwang).

### 2.5. Data Analysis

The Jadad score was used for analyzing the methodological quality of the meta-analysis and in evaluating and summarizing the 6 studies. Assessment of study quality was performed independently by two reviewers according to the Jadad scale [15]. The Jadad score is needed to ascertain the quality of clinical trials by using blind raters' assessment of the potential bias into the meta-analyses and other systematic reviews and peer review processes. The Jadad score evaluated methodologic quality by using a 5 score. The scores ranged from 0 to 5, based on randomization, blinding, allocation concealment, withdrawals, and dropouts. Studies with randomized controlled trials were considered to be of high quality if the methodological score was 3–5 points and of low quality if the methodological score was less than 3 points [15, 16].

To conduct a quantitative meta-analysis, this study performed calculations by computing the standardized difference of mean scores. This study completed all analyses using the Comprehensive Meta-Analysis program [17]. Effect size was calculated by the ratio of the mean difference between the experimental and control groups divided by the standard deviation of the control group. For example, in an evaluation with a treatment group and control group, the effect size is the difference in means between the two groups divided by the standard deviation of the control group. The effect size for cognitive therapy in children with neurodevelopmental disorder was determined using a standardized mean difference and 95% confidence intervals (CI) in a fixed-effect model, which indicates the mean improvement in standard scores of the experimental group relative to the control group. To estimate the effect size in this meta-analysis, we performed *t*-tests and *F* tests and calculated the means and standard deviations and exact *p* values.

In calculating the size of the overall effect, the sign of effect was deemed to be positive (+) when the experimental group demonstrated better effects than the control group following cognitive therapy in children with neurodevelopmental disorder and negative (−) if the converse was true. By convention, the effect size of the adaptive behavior following cognitive therapy was interpreted as large if the effect size was greater than 0.8, moderate if it was above 0.5, and small if it was above 0.2 [18]. In addition to estimating the effect size in numerical order, a forest plot was used to visually depict the estimated value of the studies' handling effect and the confidence interval of the effect size.

## 3. Results

### 3.1. General Characteristics of the Studies

The general characteristics of the six studies included in the meta-analysis are as follows. All of them were controlled trials. The methodologic quality of the primary data was assessed by using the Jadad score. This study found that 6 studies ranged from scores between 3 and 5, out of the maximum score of 5 points. The total number of participants in the studies was 544. The number of participants in each study ranged from 30 to 302. Participants in four of the studies were affected by ASD [19–22]; those in one study were diagnosed with GDD [23], and those in another singular study were diagnosed with ADHD [24]. As can be seen in Table 1, intervention of cognitive function in each of the studies included a comprehensive autism program [22], SENSE Theatre intervention [19], interpersonal synchrony [21], early start Denver model intervention [20], institutional-based therapy program [23], and visuospatial working memory training [24].

All studies used same assessment tools to evaluate adaptive behavior: Vineland Adaptive Behavior Scales [20, 22], Adaptive Behavior Assessment System [19], socially engaged imitation [21], Comprehensive Developmental Inventory for Infants and Toddlers [23], and Behavior Rating Inventory of Executive Function [24] for assessing behavior function (Table 1).

### 3.2. Effects of Cognitive Therapy on Adaptive Behavior

**Figure 2:**
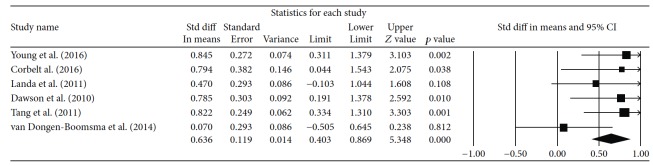
Forest plot showing individual effect sizes.

The effect sizes in this meta-analysis of studies investigating the efficacy of cognitive therapy in improving adaptive behavior ranged from −0.50 to 1.54. The overall effect size of 0.64 can be interpreted as moderate with a 95% CI (0.40, 0.87) (*p* < 0.05) (Figure 2).

### 3.3. Statistical Heterogeneity Test

The Cochran's *Q* value of 5.60 (*p* = 0.35) in our test of statistical heterogeneity indicates no significant heterogeneity. Both the fixed-effects and random-effects models showed an effect size of 0.64, providing further evidence of the lack of statistical heterogeneity among the studies included in the present meta-analysis (Table 2).

### 3.4. Publication Bias

**Figure 3:**
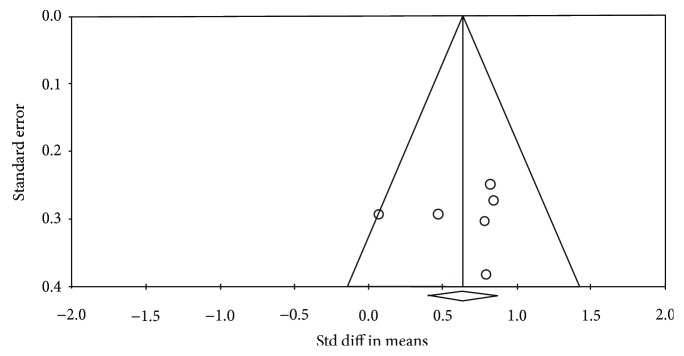
Funnel plot for publication.

Analysis of the funnel plot showed the six values to be distributed in every section, taking an asymmetric funnel shape, with more values falling on the right-hand side of the mean effect size plot. As a result, the results were considered reliable, as no significant publication bias was found (Figure 3).

## 4. Discussion

The current meta-analysis is aimed at providing a statistical synthesis of the effect on available evidence of adaptive behavior changed following cognitive therapy in children with a neurodevelopmental disorder. The overall effect size of applying cognitive therapy to address adaptive behavior with children with a neurodevelopmental disorder was found to be 0.64, a statistically significant result indicating that cognitive therapy has a moderate effect on improving adaptive behavior. No significant heterogeneity or publication bias was identified. It had adequate effect size for each study included in this meta-analysis, but the effect size by Liu et al. [25] is far from being close to an overall effect size 0.64. It may also have affected the results of a moderate effect size because few studies have utilized meta-analysis.

Based on the results of this research, it is proposed that an effective method of conducting cognitive therapy to improve adaptive behavior be developed. This meta-analysis is, so far, the first attempt to examine the empirical evidence regarding the effectiveness of cognitive therapy for adaptive behavior. The effect size of adaptive behavior with intervention of cognitive therapy found in the present investigation is larger than expected.

The results of the meta-analysis indicate that intervention of cognitive function is associated with a medium effect size, when compared with results found in the meta-analysis of the effectiveness of augmented gross motor performance in children with neurodevelopmental disorders [12]. Therefore, cognitive therapy can be used as a method of acquiring a suitable adaptive behavior to be used along with occupational therapy in a rehabilitative setting. Our research used the selected studies used in cognitive therapy to improve adaptive behavior. We are cautiously asserting that cognitive therapy is more effective than therapy of motor performance alone to improve adaptive behavior.

Intervention of cognitive function in each of the studies included a comprehensive autism program, which is used to teach communication and social behavior skills [22]. In one study, a theatre intervention was used to improve social relationships and adaptive behaviors through video modeling and concentration training by having participants visually trace from an opposite screen [19]. Interpersonal synchrony is a supplementary curriculum for social initiation, and socially synchronized engagement in toddlers is overlaid on an existing group-based comprehensive intervention [20, 21, 23, 24].

The main limitation of this meta-analysis is that many studies did not meet the inclusion criteria and therefore were excluded from the final analysis. The findings of this study have limited generalizability. Nevertheless, it has been suggested that the interpretation of the results of the meta-analysis may be valid when it is based on a data set of at least 5 studies [26]. In addition, several factors may also affect the efficacy of adaptive behavior, including such individual characteristics as types of disease, degree of cognitive and physical function, and the method of cognitive therapy. This first meta-analysis suggests that cognitive therapy improves adaptive behavior in children with neurodevelopmental disorders. A moderate effect size of cognitive therapy was identified as improving adaptive behavior in children with neurodevelopmental disorders. The results suggest that cognitive therapy is an effective clinical intervention that occupational therapists can use for children with neurodevelopmental disorders.

## Figures and Tables

**Table 1:** Characteristic of included studies.

Study	Design	Jadad score	Participants	Intervention		Outcome measure	Outcome
				Intervention/groups	Session/time		
Young et al. [22]	RCT	4	3–5-year-old students with autism spectrum disorder (*n* = 302)	(i) Comprehensive autism program (*n* = 178) (ii) Business as usual public schools (*n* = 124)	1 day per month/for 6 months	Vineland Adaptive Behavior Scales	Treatment effects were moderated by severity of ASD.
Corbett et al. [19]	RCT	3	8–14 years with autism spectrum disorder (*n* = 30)	(i) SENSE Theatre intervention (*n* = 17) (ii) Control (*n* = 13)	4 hours/10 sessions	Adaptive Behavior Assessment System	The theatre-based intervention was provided initial support for the efficacy.
Landa et al. [21]	RCT	3	Toddlers with autism spectrum disorder (*n* = 48)	(i) Interpersonal synchrony (*n* = 24) (ii) Noninterpersonal synchrony (*n* = 24)	2.5 hours per day/four days per week	Communication and Symbolic Behavior Scales Developmental Profile	A significant treatment effect was found for socially engaged imitation in the interpersonal synchrony group.
Dawson et al. [20]	RCT	3	18–30-month-old children with autism spectrum disorder (*n* = 48)	(i) Early start Denver model intervention group (*n* = 24) (ii) Assess-and-monitor group (*n* = 23)	2-hour sessions/twice per day/5 days per week/2 years	Vineland Adaptive Behavior Scales	The intervention for toddlers with ASD is for improving cognitive and adaptive behavior and reducing severity of ASD diagnosis.
Tang et al. [23]	RCT	3	Infants and toddlers with motor or global developmental delay (*n* = 70)	(i) Institutional-based therapy program (ITP) (*n* = 35) (ii) ITP plus a structured home activity program (HAPs) (*n* = 35)	(i) ITP for 45 minutes per session/12 weeks (ii) ITP 30 minutes with 15 minutes of HAPs per session/12 weeks	Comprehensive Developmental Inventory for Infants and Toddlers	Early intervention programs are helpful for these children.
van Dongen-Boomsma et al. [24]	RCT	5	5–7-year-old children with attention-deficit/hyperactivity disorder	(i) Visuospatial working memory group (*n* = 26) (ii) placebo group (*n* = 21)	15 minutes/5 days a week/25 sessions	Behavior Rating Inventory of Executive Function	No significant treatment effect on any of the primary or other secondary outcome measurements was found.

**Table 2:** Heterogeneity.

Model	Effect size	*p* value	*Q* value	*p* value
Fixed	0.64	0.00	5.60	0.35
Random	0.64	0.00		
